# *catena*-Poly[[[aqua­copper(II)]-bis­[μ-bis­(3,5-dimethyl-1*H*-pyrazol-4-yl) selenide-κ^2^ *N* ^2^:*N* ^2′^]] dichloride monohydrate]

**DOI:** 10.1107/S1600536810007403

**Published:** 2010-03-03

**Authors:** Maksym Seredyuk, Yurii S. Moroz, Kateryna O. Znovjyak, Vadim A. Pavlenko, Igor O. Fritsky

**Affiliations:** a Department of Chemistry, National Taras Shevchenko University, Volodymyrska Street 64, 01601 Kyiv, Ukraine

## Abstract

In the title compound, {[Cu(C_10_H_14_N_4_Se)_2_(H_2_O)]Cl_2_·H_2_O}_*n*_, the Cu^II^ ion, lying on a twofold rotation axis, has a square-pyramidal geometry constituted by four N atoms of pyrazolyl groups in the basal plane and an apical O atom of a water mol­ecule. A pair of bis­(3,5-dimethyl-1*H*-pyrazol-4-yl) selenide ligands bridge the Cu centers into a polymeric double-chain extending along [001]. The chloride anions are involved in inter­molecular N—H⋯Cl and O—H⋯Cl hydrogen bonds, which link the chains into a three-dimensional network.

## Related literature

For general background to the applications of coordination polymers, see: Farha *et al.* (2009 ▶); Shibahara *et al.* (2007 ▶); Zhang *et al.* (2009 ▶). For our studies of similar complexes, see: Seredyuk *et al.* (2007 ▶, 2009 ▶). 
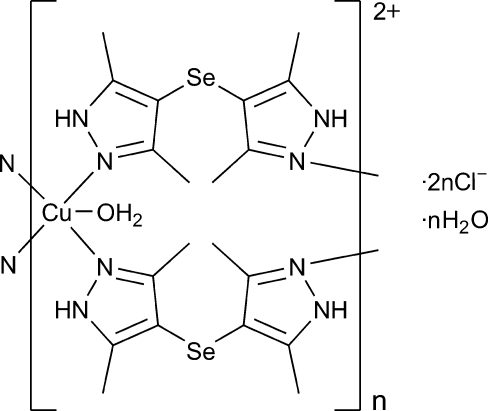


## Experimental

### 

#### Crystal data

[Cu(C_10_H_14_N_4_Se)_2_(H_2_O)]Cl_2_·H_2_O
*M* *_r_* = 708.90
Monoclinic, 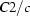

*a* = 11.332 (1) Å
*b* = 13.229 (2) Å
*c* = 18.786 (1) Å
β = 92.45 (3)°
*V* = 2813.7 (5) Å^3^
*Z* = 4
Mo *K*α radiation
μ = 3.59 mm^−1^
*T* = 100 K
0.10 × 0.05 × 0.01 mm

#### Data collection

Kuma KM-4 CCD diffractometer
6625 measured reflections
2377 independent reflections
2217 reflections with *I* > 2σ(*I*)
*R*_int_ = 0.061

#### Refinement

*R*[*F* ^2^ > 2σ(*F* ^2^)] = 0.026
*wR*(*F* ^2^) = 0.072
*S* = 1.10
2377 reflections
164 parameters
H-atom parameters constrained
Δρ_max_ = 0.57 e Å^−3^
Δρ_min_ = −0.44 e Å^−3^

### 

Data collection: *KM-4 CCD Software.* (Kuma Diffraction, 1998 ▶); cell refinement: *KM-4 CCD Software.*; data reduction: *KM-4 CCD Software.*; program(s) used to solve structure: *SHELXS97* (Sheldrick, 2008 ▶); program(s) used to refine structure: *SHELXL97* (Sheldrick, 2008 ▶); molecular graphics: *ORTEP-3* (Farrugia, 1997 ▶); software used to prepare material for publication: *WinGX* (Farrugia, 1999 ▶).

## Supplementary Material

Crystal structure: contains datablocks I, global. DOI: 10.1107/S1600536810007403/hy2286sup1.cif

Structure factors: contains datablocks I. DOI: 10.1107/S1600536810007403/hy2286Isup2.hkl

Additional supplementary materials:  crystallographic information; 3D view; checkCIF report

## Figures and Tables

**Table 1:** Hydrogen-bond geometry (Å, °)

*D*—H⋯*A*	*D*—H	H⋯*A*	*D*⋯*A*	*D*—H⋯*A*
O1*W*—H1*W*⋯Cl1	0.93	2.43	3.354 (2)	169
O1—H1*O*1⋯Cl1	0.88	2.25	3.0702 (10)	156
N2—H2*N*⋯Cl1^i^	0.88	2.33	3.117 (2)	148
N4—H4*N*⋯Cl1^ii^	0.88	2.27	3.144 (2)	176

Symmetry codes: (i) 
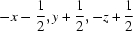
; (ii) 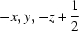.

**Fig. 1.**
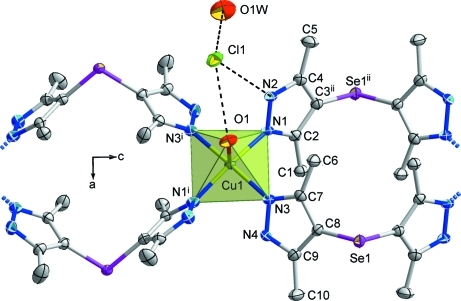
A portion of the double-chain structure of the title compound, showing the 50% probability displacement ellipsoids. H atoms are omitted for clarity. Dashed lines denote hydrogen bonds. [Symmetry codes: (i) -x, y, 1/2-z; (ii) -x, -y, 1-z.]

**Fig. 2.**
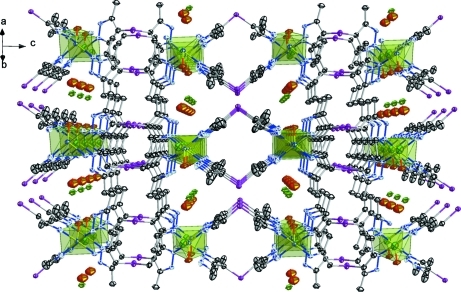
A packing diagram of the title compound. H atoms are omitted for clarity.

## supplementary crystallographic information

### Comment

Study of metal-organic polymers is a well elaborated research area in coordination chemistry. Infinite molecular polymeric arrays are potentially applicable as specifically ordered crystalline substances with reversible selective sorption (Farha *et al.*, 2009; Zhang *et al.*, 2009), electrical conductivity (Zhang *et al.*, 2009) and molecular magnetism functionality (Shibahara *et al.*, 2007).

The title compound was prepared in a water–methanolic medium by mixing solutions of CuCl_2_.2H_2_O and the bis(3,5-dimethyl-1*H*-pyrazolyl)selenide (*L*) ligand. It is similar to the copper compounds reported recently (Seredyuk *et al.*, 2007, 2009). A square pyramidal environment of the Cu^II^ ion is constituted by four non-coplanar N atoms of pyrazolyl rings [the Cu—N distances are 1.988 (2) and 2.017 (2) Å, the Cu—O distance is 2.208 (3) Å]. Adjacent Cu^II^ ions are linked by symmetrically equivalent ligands in a double-stranded bridge fashion (Fig. 1). Formed one-dimensional linear chain is running along the *c* axis, where the Cu atom deviates from the average basal plane by a value of 0.392 (1) Å (Fig. 2). The NH group of a pyrazole ring is involved in hydrogen bonding with chloride anion (Table 1), which further forms hydrogen bonds with both free and coordinated water molecules and additionally with a pyrazole ring of a neighbouring polymeric chain (Table 1). As a result, a dense network of hydrogen bonds is formed.

### Experimental

The ligand *L* was prepared according to a previously reported method (Seredyuk *et al.*, 2007). Copper(II) chloride dihydrate (0.034 g, 0.19 mmol) in water (5 ml) was added to 5 ml of hot methanol solution of *L* (0.100 g, 0.37 mmol). The solution was left for slow cooling at room temperature. After several days plate-like blue-violet crystals of the title compound suitable for X-ray analysis were isolated. Analysis, calculated for C_20_H_32_Cl_2_CuN_8_O_2_Se_2_: C 33.89, H 4.55, N 15.81%; found: C 33.67, H 4.51, N 15.60%.

### Refinement

C- and N-bound H atoms were placed at calculated positions and treated as riding on their parent atoms [C—H = 0.98 Å and *U*_iso_(H) = 1.5*U*_eq_(C); N—H = 0.88 Å and *U*_iso_(H) = 1.2*U*_eq_(N)]. The H atoms of water molecules were located from a difference Fourier map and were refined as riding, with *U*_iso_(H) = 1.5*U*_eq_(O).

### Crystal data

**Table:**

[Cu(C_10_H_14_N_4_Se)_2_(H_2_O)]Cl_2_·H_2_O	*F*(000) = 1420
*M**_r_* = 708.90	*D*_x_ = 1.673 Mg m^−^^3^
Monoclinic, *C*2/*c*	Mo *K*α radiation, λ = 0.71073 Å
Hall symbol: -C 2yc	Cell parameters from 6625 reflections
*a* = 11.332 (1) Å	θ = 3.2–28.4°
*b* = 13.229 (2) Å	µ = 3.59 mm^−^^1^
*c* = 18.786 (1) Å	*T* = 100 K
β = 92.45 (3)°	Plates, blue
*V* = 2813.7 (5) Å^3^	0.10 × 0.05 × 0.01 mm
*Z* = 4	

### Data collection

**Table:**

Kuma KM-4 CCD diffractometer	2217 reflections with *I* > 2σ(*I*)
Radiation source: fine-focus sealed tube	*R*_int_ = 0.061
graphite	θ_max_ = 25.0°, θ_min_ = 3.2°
ω scans	*h* = −13→7
6625 measured reflections	*k* = −15→15
2377 independent reflections	*l* = −22→22

### Refinement

**Table:**

Refinement on *F*^2^	Primary atom site location: structure-invariant direct methods
Least-squares matrix: full	Secondary atom site location: difference Fourier map
*R*[*F*^2^ > 2σ(*F*^2^)] = 0.026	Hydrogen site location: inferred from neighbouring sites
*wR*(*F*^2^) = 0.072	H-atom parameters constrained
*S* = 1.10	*w* = 1/[σ^2^(*F*_o_^2^) + (0.0419*P*)^2^ + 1.7165*P*] where *P* = (*F*_o_^2^ + 2*F*_c_^2^)/3
2377 reflections	(Δ/σ)_max_ < 0.001
164 parameters	Δρ_max_ = 0.57 e Å^−^^3^
0 restraints	Δρ_min_ = −0.44 e Å^−^^3^

### Fractional atomic coordinates and isotropic or equivalent isotropic displacement parameters (Å^2^)

**Table:**

	*x*	*y*	*z*	*U*_iso_*/*U*_eq_	
C1	−0.0470 (2)	−0.1461 (2)	0.41623 (14)	0.0171 (6)	
H22A	0.0111	−0.1105	0.4471	0.026*	
H22B	−0.0068	−0.1790	0.3773	0.026*	
H22C	−0.0873	−0.1974	0.4440	0.026*	
C2	−0.1350 (2)	−0.07273 (19)	0.38631 (13)	0.0112 (5)	
C3	0.2414 (2)	0.04235 (18)	0.58358 (13)	0.0106 (5)	
C4	−0.2939 (2)	0.02624 (19)	0.36872 (14)	0.0137 (5)	
C5	−0.4086 (2)	0.0822 (2)	0.36926 (16)	0.0232 (7)	
H9A	−0.4637	0.0538	0.3330	0.035*	
H9B	−0.3950	0.1538	0.3589	0.035*	
H9C	−0.4421	0.0758	0.4163	0.035*	
C6	0.0587 (2)	0.14112 (19)	0.38756 (14)	0.0124 (5)	
H5A	0.0236	0.1593	0.3407	0.019*	
H5B	0.1033	0.1988	0.4074	0.019*	
H5C	−0.0040	0.1230	0.4196	0.019*	
C7	0.1401 (2)	0.05297 (19)	0.37995 (13)	0.0106 (5)	
C8	0.2365 (2)	0.02232 (19)	0.42508 (13)	0.0115 (5)	
C9	0.2796 (2)	−0.0650 (2)	0.39493 (13)	0.0129 (5)	
C10	0.3787 (2)	−0.1344 (2)	0.41780 (15)	0.0205 (6)	
H14A	0.3463	−0.1955	0.4392	0.031*	
H14B	0.4311	−0.1001	0.4529	0.031*	
H14C	0.4234	−0.1532	0.3763	0.031*	
N1	−0.12324 (17)	−0.02426 (15)	0.32427 (11)	0.0112 (4)	
N2	−0.22165 (18)	0.03492 (15)	0.31502 (11)	0.0128 (4)	
H2N	−0.2356	0.0742	0.2778	0.015*	
N3	0.12555 (17)	−0.01244 (16)	0.32624 (11)	0.0113 (4)	
N4	0.21226 (19)	−0.08360 (16)	0.33652 (12)	0.0123 (5)	
H4N	0.2223	−0.1354	0.3080	0.015*	
O1	0.0000	−0.19654 (18)	0.2500	0.0154 (5)	
H1O1	−0.0625	−0.2317	0.2606	0.023*	
O1W	−0.5000	−0.1193 (3)	0.2500	0.0387 (8)	
H1W	−0.4338	−0.1589	0.2619	0.058*	
Cl1	−0.25642 (5)	−0.26253 (5)	0.26925 (3)	0.01824 (17)	
Cu1	0.0000	−0.02962 (3)	0.2500	0.00888 (13)	
Se1	0.30884 (2)	0.097275 (18)	0.501388 (12)	0.01122 (11)	

### Atomic displacement parameters (Å^2^)

**Table:**

	*U*^11^	*U*^22^	*U*^33^	*U*^12^	*U*^13^	*U*^23^
C1	0.0139 (12)	0.0217 (15)	0.0161 (14)	0.0071 (11)	0.0043 (11)	0.0059 (10)
C2	0.0097 (11)	0.0122 (12)	0.0116 (13)	−0.0016 (10)	−0.0015 (10)	−0.0020 (10)
C3	0.0099 (11)	0.0106 (12)	0.0114 (13)	−0.0014 (9)	0.0035 (10)	−0.0007 (9)
C4	0.0112 (12)	0.0169 (14)	0.0134 (14)	0.0001 (10)	0.0038 (11)	−0.0019 (10)
C5	0.0174 (14)	0.0321 (17)	0.0204 (15)	0.0133 (12)	0.0048 (12)	0.0067 (12)
C6	0.0120 (12)	0.0111 (13)	0.0139 (13)	0.0018 (10)	0.0005 (10)	−0.0001 (9)
C7	0.0087 (11)	0.0117 (12)	0.0113 (13)	−0.0023 (10)	0.0020 (10)	0.0011 (9)
C8	0.0077 (11)	0.0162 (13)	0.0105 (13)	−0.0012 (10)	0.0007 (10)	−0.0004 (9)
C9	0.0091 (12)	0.0158 (13)	0.0138 (14)	0.0009 (10)	0.0004 (11)	−0.0009 (10)
C10	0.0153 (13)	0.0217 (15)	0.0242 (15)	0.0091 (11)	−0.0030 (12)	−0.0013 (11)
N1	0.0090 (10)	0.0106 (11)	0.0139 (11)	0.0019 (8)	−0.0005 (9)	−0.0001 (8)
N2	0.0118 (10)	0.0162 (11)	0.0103 (11)	0.0042 (9)	−0.0009 (9)	0.0022 (8)
N3	0.0069 (10)	0.0116 (11)	0.0154 (11)	0.0024 (8)	0.0013 (9)	0.0009 (8)
N4	0.0108 (10)	0.0126 (11)	0.0134 (11)	0.0041 (8)	0.0004 (9)	−0.0020 (8)
O1	0.0122 (12)	0.0104 (13)	0.0241 (14)	0.000	0.0053 (11)	0.000
O1W	0.0318 (17)	0.0365 (19)	0.048 (2)	0.000	0.0058 (16)	0.000
Cl1	0.0215 (3)	0.0131 (3)	0.0206 (4)	−0.0055 (2)	0.0061 (3)	−0.0008 (2)
Cu1	0.0069 (2)	0.0108 (2)	0.0089 (2)	0.000	0.00069 (17)	0.000
Se1	0.00924 (16)	0.01421 (17)	0.01027 (17)	−0.00342 (9)	0.00104 (11)	−0.00041 (8)

### Geometric parameters (Å, °)

**Table:**

C1—C2	1.485 (3)	C7—N3	1.334 (3)
C1—H22A	0.9800	C7—C8	1.413 (4)
C1—H22B	0.9800	C8—C9	1.385 (4)
C1—H22C	0.9800	C8—Se1	1.900 (2)
C2—N1	1.342 (3)	C9—N4	1.332 (3)
C2—C3^i^	1.412 (3)	C9—C10	1.499 (3)
C3—C4^i^	1.391 (4)	C10—H14A	0.9800
C3—C2^i^	1.412 (3)	C10—H14B	0.9800
C3—Se1	1.896 (2)	C10—H14C	0.9800
C4—N2	1.331 (3)	N1—N2	1.368 (3)
C4—C3^i^	1.391 (4)	N2—H2N	0.8800
C4—C5	1.496 (4)	N3—N4	1.368 (3)
C5—H9A	0.9800	N4—H4N	0.8800
C5—H9B	0.9800	O1—H1O1	0.8771
C5—H9C	0.9800	O1W—H1W	0.9337
C6—C7	1.497 (3)	Cu1—N1^ii^	2.017 (2)
C6—H5A	0.9800	Cu1—N3^ii^	1.988 (2)
C6—H5B	0.9800	Cu1—O1	2.208 (3)
C6—H5C	0.9800		
			
C2—C1—H22A	109.5	N4—C9—C8	106.9 (2)
C2—C1—H22B	109.5	N4—C9—C10	121.2 (2)
H22A—C1—H22B	109.5	C8—C9—C10	131.8 (2)
C2—C1—H22C	109.5	C9—C10—H14A	109.5
H22A—C1—H22C	109.5	C9—C10—H14B	109.5
H22B—C1—H22C	109.5	H14A—C10—H14B	109.5
N1—C2—C3^i^	109.3 (2)	C9—C10—H14C	109.5
N1—C2—C1	123.4 (2)	H14A—C10—H14C	109.5
C3^i^—C2—C1	127.3 (2)	H14B—C10—H14C	109.5
C4^i^—C3—C2^i^	106.1 (2)	C2—N1—N2	105.85 (19)
C4^i^—C3—Se1	126.76 (19)	C2—N1—Cu1	132.97 (17)
C2^i^—C3—Se1	126.70 (19)	N2—N1—Cu1	121.14 (15)
N2—C4—C3^i^	106.5 (2)	C4—N2—N1	112.3 (2)
N2—C4—C5	121.7 (2)	C4—N2—H2N	123.9
C3^i^—C4—C5	131.8 (2)	N1—N2—H2N	123.9
C4—C5—H9A	109.5	C7—N3—N4	105.95 (19)
C4—C5—H9B	109.5	C7—N3—Cu1	132.87 (17)
H9A—C5—H9B	109.5	N4—N3—Cu1	120.69 (16)
C4—C5—H9C	109.5	C9—N4—N3	111.8 (2)
H9A—C5—H9C	109.5	C9—N4—H4N	124.1
H9B—C5—H9C	109.5	N3—N4—H4N	124.1
C7—C6—H5A	109.5	Cu1—O1—H1O1	122.0
C7—C6—H5B	109.5	N3^ii^—Cu1—N3	166.88 (12)
H5A—C6—H5B	109.5	N3^ii^—Cu1—N1^ii^	89.60 (8)
C7—C6—H5C	109.5	N3—Cu1—N1^ii^	89.94 (8)
H5A—C6—H5C	109.5	N3^ii^—Cu1—N1	89.94 (8)
H5B—C6—H5C	109.5	N3—Cu1—N1	89.60 (8)
N3—C7—C8	109.6 (2)	N1^ii^—Cu1—N1	175.97 (11)
N3—C7—C6	121.4 (2)	N3^ii^—Cu1—O1	96.56 (6)
C8—C7—C6	129.0 (2)	N3—Cu1—O1	96.56 (6)
C9—C8—C7	105.7 (2)	N1^ii^—Cu1—O1	92.01 (6)
C9—C8—Se1	126.54 (19)	N1—Cu1—O1	92.01 (6)
C7—C8—Se1	126.90 (19)	C3—Se1—C8	103.80 (10)
			
N3—C7—C8—C9	0.5 (3)	C10—C9—N4—N3	178.6 (2)
C6—C7—C8—C9	178.4 (2)	C7—N3—N4—C9	0.4 (3)
N3—C7—C8—Se1	170.61 (17)	Cu1—N3—N4—C9	−172.60 (17)
C6—C7—C8—Se1	−11.5 (4)	C7—N3—Cu1—N3^ii^	38.4 (2)
C7—C8—C9—N4	−0.2 (3)	N4—N3—Cu1—N3^ii^	−150.87 (17)
Se1—C8—C9—N4	−170.41 (18)	C7—N3—Cu1—N1^ii^	126.3 (2)
C7—C8—C9—C10	−178.7 (3)	N4—N3—Cu1—N1^ii^	−62.89 (17)
Se1—C8—C9—C10	11.1 (4)	C7—N3—Cu1—N1	−49.7 (2)
C3^i^—C2—N1—N2	−0.7 (3)	N4—N3—Cu1—N1	121.11 (17)
C1—C2—N1—N2	179.3 (2)	C7—N3—Cu1—O1	−141.6 (2)
C3^i^—C2—N1—Cu1	−178.10 (17)	N4—N3—Cu1—O1	29.13 (17)
C1—C2—N1—Cu1	2.0 (4)	C2—N1—Cu1—N3^ii^	143.2 (2)
C3^i^—C4—N2—N1	−0.1 (3)	N2—N1—Cu1—N3^ii^	−33.83 (17)
C5—C4—N2—N1	−178.6 (2)	C2—N1—Cu1—N3	−49.9 (2)
C2—N1—N2—C4	0.5 (3)	N2—N1—Cu1—N3	133.06 (17)
Cu1—N1—N2—C4	178.26 (17)	C2—N1—Cu1—O1	46.7 (2)
C8—C7—N3—N4	−0.5 (3)	N2—N1—Cu1—O1	−130.39 (16)
C6—C7—N3—N4	−178.6 (2)	C4^i^—C3—Se1—C8	102.0 (2)
C8—C7—N3—Cu1	171.23 (17)	C2^i^—C3—Se1—C8	−87.0 (2)
C6—C7—N3—Cu1	−6.9 (4)	C9—C8—Se1—C3	−92.4 (2)
C8—C9—N4—N3	−0.1 (3)	C7—C8—Se1—C3	99.4 (2)

Symmetry codes: (i) −*x*, −*y*, −*z*+1; (ii) −*x*, *y*, −*z*+1/2.

### Hydrogen-bond geometry (Å, °)

**Table:**

*D*—H···*A*	*D*—H	H···*A*	*D*···*A*	*D*—H···*A*
O1W—H1W···Cl1	0.93	2.43	3.354 (2)	169
O1—H1O1···Cl1	0.88	2.25	3.0702 (10)	156
N2—H2N···Cl1^iii^	0.88	2.33	3.117 (2)	148
N4—H4N···Cl1^ii^	0.88	2.27	3.144 (2)	176

Symmetry codes: (iii) −*x*−1/2, *y*+1/2, −*z*+1/2; (ii) −*x*, *y*, −*z*+1/2.
